# Homocysteine is a risk factor for reduced ejection fraction in children with myocarditis: a single-center study

**DOI:** 10.3389/fped.2026.1666056

**Published:** 2026-02-25

**Authors:** Chengjun Zhang, Xiuwen Ren, Yufan Xu, Yun Dong, Yi Li, Xi Yang, Guiying Liu

**Affiliations:** 1 Department of Pediatric, Beijing Anzhen Hospital, Capital Medical University, Beijing, China; 2 School of Public Health, Capital Medical University, Beijing, China

**Keywords:** cardiac magnetic resonance, cardiac MRI, ejection fraction, homocysteine, myocarditis, pediatrics

## Abstract

**Background:**

The relationship between homocysteine (HCY) and ejection fraction (EF) has been demonstrated in diseases such as coronary artery disease, but the relationship between HCY and EF in pediatric patients with myocarditis remains unclear. The aim of this study was to investigate the relationship between HCY and EF in pediatric patients with myocarditis.

**Methods:**

This single-center cross-sectional study included 164 pediatric myocarditis patients aged 1–18 years, including 104 males and 60 females, at Anzhen Hospital (2023–2024) in Beijing. Patient demographic characteristics were collected, and blood tests were performed to assess HCY, routine blood tests, and markers of myocardial damage. EF was measured using 3.0T cardiac magnetic resonance (CMR), and patients were grouped using EF < 55% as the cutoff value. Statistical analyses were performed using t-tests, Binary logistic regression and Restrict cubic spline (RCS), and subgroup analyses [age, sex, body mass index (BMI)].

**Results:**

Of the 164 patients, 31% (*n* = 51) had EF values < 55%. High HCY concentration demonstrated a statistically positive relationship with the risk of occurrence of EF < 55% (OR = 1.033, *P* = 0.034). Subgroup analysis showed a stronger correlation in men (OR = 1.045, *P* = 0.016) and in those with a BMI ≥ 24 kg/m^2^ (OR = 1.083, *P* = 0.010). The RCS showed a non-significant trend of increasing EF < 55% (*P* > 0.05).

**Conclusion:**

The findings suggest that elevated HCY levels are a risk factor for EF < 55% in pediatric patients with myocarditis, especially in males and overweight individuals.

## 1 Introduction

Homocysteine (HCY) is a sulfhydryl-containing amino acid that occupies a pivotal role in one-carbon metabolism, functioning as a critical intermediate in two interconnected pathways: remethylation to methionine and transulfuration to cysteine (1, 2). Elevated plasma HCY levels, termed hyperhomocysteinemia, arise from a combination of genetic predispositions [e.g., mutations in methylenetetrahydrofolate reductase (MTHFR) or cystathionine *β*-synthase (CBS) genes], nutritional deficiencies (particularly folate, vitamin B6, or vitamin B12), and impaired renal clearance, which is a common contributor in chronic kidney disease (3). Accumulating evidence underscores hyperhomocysteinemia as a robust biomarker for cardiovascular pathology. Specifically, elevated HCY levels have been mechanistically linked to endothelial dysfunction via oxidative stress-mediated depletion of nitric oxide bioavailability, vascular inflammation through NF-*κ*B activation and pro-inflammatory cytokine release, and thrombogenesis due to enhanced platelet aggregation and impaired anticoagulant pathways (4). These mechanisms collectively contribute to the pathogenesis of atherosclerosis, hypertension, and ischemic heart disease (5). Furthermore, clinical studies highlight a dose-dependent relationship between HCY levels and cardiovascular mortality, independent of traditional risk factors, solidifying its role as both a diagnostic and prognostic marker in cardiovascular medicine (6, 7).

Ejection fraction (EF) is a critical indicator for assessing cardiac systolic function, typically measured by echocardiography (8), with cardiac magnetic resonance (CMR) providing more accurate EF measurements (9, 10). Research indicates that when EF falls below 55%, patients experience significantly increased mortality and cardiovascular event risks (11). Furthermore, EF changes are closely related to prognosis, as an EF below 55% typically reflects substantial impairment of cardiac pumping capacity, potentially exacerbating heart failure symptoms and reducing quality of life (11). Consequently, a clinical threshold of 55% EF is commonly used to distinguish populations with normal EF from those with mildly abnormal cardiac function (12).

Myocarditis is clinically and pathologically defined as an inflammatory disease characterized by pathological changes predominantly confined to the myocardium, which can lead to cardiac dysfunction and ventricular remodeling (13, 14). Its etiology primarily includes infectious factors, such as viral and bacterial infections, and non-infectious causes, such as autoimmune diseases (15). In the pediatric population, myocarditis continues to exhibit high morbidity and significant disease burden. A nationwide epidemiological survey of hospitalized children in China revealed an increasing prevalence of myocarditis among pediatric inpatients, with a mortality rate of 4.13% (16).

Patients with severe myocarditis may develop ventricular remodeling, leading to a decrease in EF. The change in EF measured by the initial CMR performed early after acute myocarditis onset is an independent predictor of adverse outcomes in acute myocarditis (17, 18). Multiple studies have confirmed an inverse correlation between HCY levels and EF in patients with coronary artery disease, diabetes, and chronic heart failure (19–21). Nygard et al. (1997) observed in patients with coronary artery disease that elevated HCY levels were significantly associated with reduced LVEF, with a significantly higher mortality rate in the high HCY group (≥ 15 μmol/L), which suggests that HCY is not only a marker of cardiac insufficiency, but also an independent prognostic predictor (22). A negative correlation between HCY and LVEF was also identified in patients undergoing coronary artery bypass grafting (CABG) surgery (23). A study by El-Amrousy et al. (2017) reporting an association between HCY and EF in pediatric acute heart failure, no study to date has specifically examined the association between HCY levels and reduced EF in children diagnosed with myocarditis using CMR imaging (24). Given the unique pathophysiological features of myocarditis and the enhanced accuracy of CMR for evaluating left ventricular function, this study addresses a critical gap in the pediatric cardiovascular literature. This study aims to investigate the association between elevated HCY levels and reduced EF in pediatric patients with myocarditis, explore the dose-response relationship between HCY concentration and the risk of reduced EF, and examine whether this association varies across clinically relevant subgroups stratified by age, sex, and body mass index (BMI). We hypothesize that elevated plasma HCY levels are associated with an increased risk of reduced EF (EF < 55%) in pediatric patients with myocarditis.

## 2 Methods

### 2.1 Study participants

We performed a single-center cross-sectional study involving 350 hospitalized patients admitted to Department of General Pediatrics in Anzhen Hospital in Beijing, China from January 2023 to January 2024. Inclusion criteria: (1) diagnosis consistent with the “Diagnostic recommendation for myocarditis in children (version 2018)” (14): A clinical diagnosis of myocarditis can be made when ≥ 3 major clinical diagnostic criteria are met, or when 2 major criteria plus ≥ 3 minor criteria are present, and other diseases are excluded.

(I) Major Diagnostic Criteria
  1 Cardiac dysfunction, cardiogenic shock, or cardio-cerebral syndrome.
  2 Cardiac enlargement.
  3 Elevated serum cardiac troponin T or I (cTnI or cTnT) or creatine kinase MB subunit (CK-MB) with dynamic changes.
  4 Significant electrocardiographic (ECG) changes (ECG or 24-hour Holter monitoring). Includes: ST-T segment changes in two or more primary leads (I, II, aVF, V5) dominated by R waves persisting for more than 4 days with dynamic variation; newly identified sinoatrial or atrioventricular block; complete right or left bundle branch block; sinus arrest; pairs, polymorphic, or multifocal premature contractions, ectopic tachycardia not caused by the AV node or AV reentrant circuits, atrial flutter, atrial fibrillation, ventricular flutter, ventricular fibrillation, low-voltage QRS complexes (excluding neonates), or abnormal Q waves.
  5 CMR demonstrates typical myocardial inflammatory features. At least two of the following three criteria must be present: (1) Myocardial edema: focal or diffuse hyperintensity on T2-weighted images; (2) myocardial hyperemia and capillary leakage: early gadolinium enhancement on T1-weighted images; (3) Myocardial necrosis and fibrosis: At least one focal area of late gadolinium enhancement on T1-weighted images, distributed in non-ischemic regions.
(II) Secondary Clinical Diagnostic Criteria
  (a) History of preceding infection, such as upper respiratory or gastrointestinal viral infection within 1–3 weeks prior to onset.
  (b) 2. Symptoms including chest tightness, chest pain, palpitations, fatigue, dizziness, pallor, grayish complexion, or abdominal pain (at least 2 items).
  (c) Elevated serum lactate dehydrogenase (LDH), *α*-hydroxybutyrate dehydrogenase (*α*-HBDH), or aspartate transaminase (AST).
  (d) Mild electrocardiogram (ECG) abnormalities. This refers to ST-T changes that do not meet the criteria for “significant ECG changes” in the primary clinical diagnostic criteria for myocarditis.
  (e) Positive anti-myocardial antibodies.

**Figure 1:**
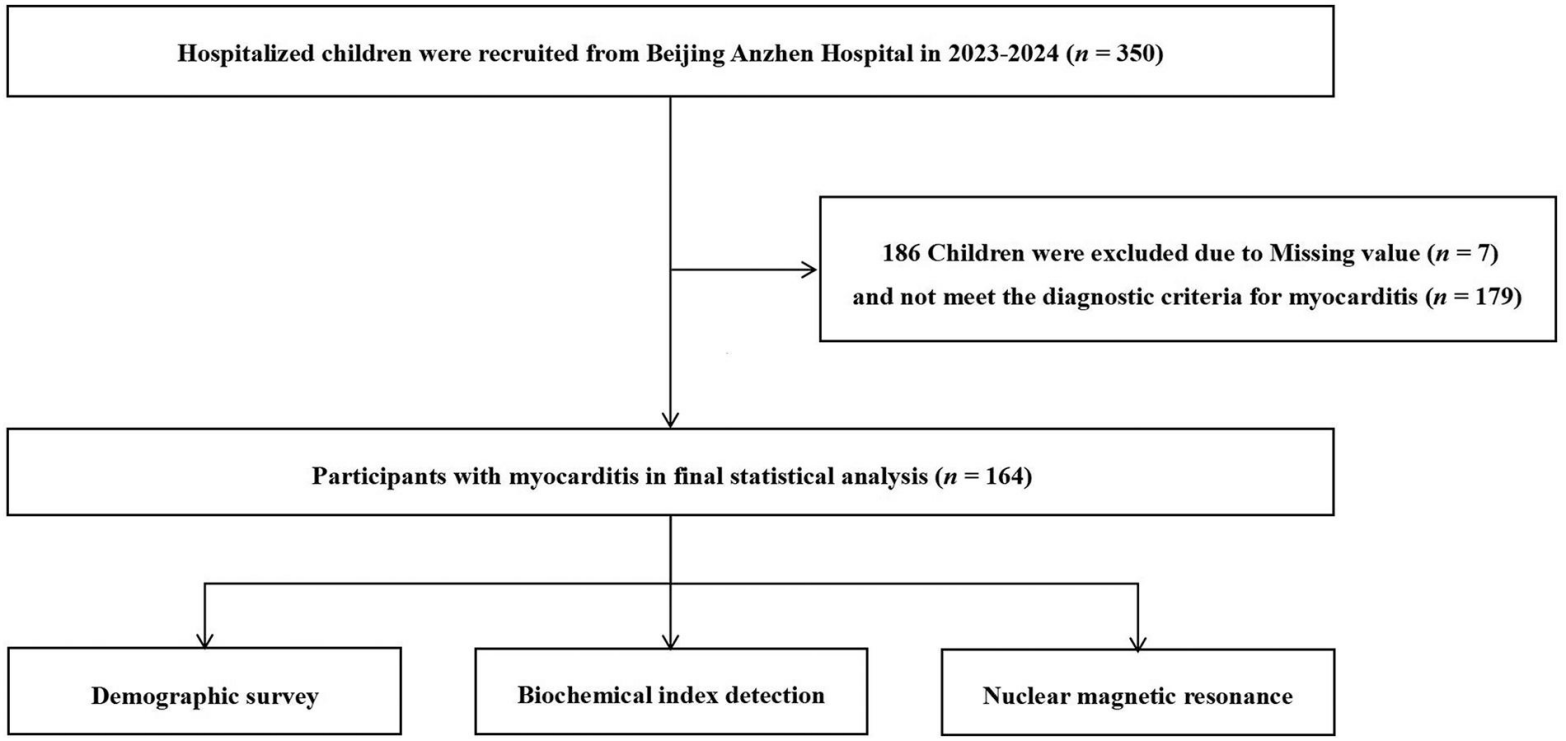
Flow chart of the study participants selection process.

(2) 1 year < age < 18 years; (3) informed consent from the guardians. Exclusion criteria: (1) other cardiovascular diseases, such as coronary artery disease, congenital heart disease, cardiomyopathies and congenital atrioventricular block; (2) metabolic disorders, including hyperthyroidism and other inherited metabolic diseases; (3) other disease such as ion channelopathies, orthostatic intolerance, beta-receptor hyperactivity, and drug-induced electrocardiographic changes. (4) missing value.186 pediatric patients were excluded due to missing value (*n* = 7) and not meet the diagnostic criteria for myocarditis (*n* = 179). Ultimately, 164 subjects were included in the final statistical analysis (Figure 1). This study was conducted in accordance with the Declaration of Helsinki and has been approved by the Ethics Committee of Beijing Anzhen Hospital (No.2025072x) on March 20, 2025. Written informed consent were obtained from all pediatric patients and their legal guardians before participation in the study.

### 2.2 Sample collection and laboratory analysis

All participants conducted routine examinations upon admission, including collection of demographic data, anthropometric measures (height and weight) and BMI was calculated as weight (kg)/height (m^2^). Serum biomarkers including white blood cell count (WBC), C-reactive protein (CRP), hemoglobin (HGB), platelet count (PLT), interleukin-6 (IL-6), creatine kinase (CK), creatine kinase-MB isoenzyme (CK-MB), troponin I (TnI), N-terminal pro-B-type natriuretic peptide (NT-proBNP), folate, vitamin B12, and the percentage of band neutrophils and segmented neutrophils were analyzed using standard laboratory methods. Serum HCY was measured using enzymatic cycling assay on DIASYS diagnostic systems (Shanghai, China). The reference range for HCY is 3–50 μmol/L. The intra-/inter-assay coefficient of variation (CV) is 3.02% and 4.81%, respectively. Currently, there is no established specific reference range for plasma HCY in children in China, and the reference range varies depending on age and detection methods (25). In the present study, the normal values of HCY under our measurement method were 6–14 μmol/L.

All pediatric patients completed CMR (3.0T, Discovery MR750, GE, USA) within 72 h of admission. The sequences types include TrueFisp; turboFlash; myocardial resting perfusion imaging; myocardial delayed imaging sequences; cine MRI; TSE, T1WI, T2WI. After scout scanning, cine sequences were acquired to assess cardiac function. Left ventricular end-diastolic volume (LVEDV), EF, and systolic wall thickness (SWT) were measured. software used for EF was cvi42, version 5.17; Circle Cardiovascular Imaging. T2-weighted imaging was performed using a triple inversion recovery sequence to capture extent of high-signal areas within myocardial tissue, reflecting the presence of myocardial edema. The ratio of myocardial signal intensity to skeletal muscle signal intensity at the same level was calculated, with a ratio exceeding 2.0 indicating edema. The presence and distribution of myocardial edema were recorded according to 17-segment of the left ventricle.Subsequently, inject the contrast agent Gd- using a high-pressure injector. Following contrast administration, Early gadolinium enhancement (EGE) imaging was performed using a triple inversion recovery sequence. The extent of early enhancement was assessed by comparison with the signal intensity of adjacent skeletal muscle. Myocarditis was considered when the whole myocardial-to-skeletal muscle signal intensity ratio was ≥ 4.0 or when the absolute extent of myocardial enhancement was ≥ 45%.Late gadolinium enhancement (LGE) imaging was subsequently acquired 5–10 min after contrast administration using the same triple inversion recovery sequence. The presence of LGE was considered indicative of myocardial fibrosis or necrosis. The extent, frequency, and degree of myocardial involvement of LGE were recorded. When LGE demonstrated a subendocardial distribution corresponding to a coronary artery territory, myocardial infarction was considered, and such cases were excluded from the diagnosis of myocarditis.

This study employed a self-designed data collection form to systematically document each child with myocarditis’ baseline status, serum biomarkers, and relevant CMR examination details.

### 2.3 Statistical analysis

SPSS 26.0 and R 4.2.3 were used for data analysis. Graphs were drafted by Graph pad Prism 8 and R 4.2.3. Quantitative data was expressed as mean ± SD. Categorical data was expressed as number and percentage [*n* (%)]. The normal distribution test using SPSS. Although HCY values were not normally distributed based on the Shapiro–Wilk test, the sample size in each group exceeded 50, and logistic regression is robust to moderate violations of normality. Therefore, no data transformation was applied. Independent samples t-test and *χ*^2^ test was used to compare the differences between two groups. General linear model (GLM) was used to compare the differences of biochemical parameters between groups. Binary logistic regression and restrict cubic spline (RCS) were conducted to explore the association between plasma parameters and the risk of the occurrence of EF < 55%. Confounding factors, including age, gender and BMI were adjusted during analysis. A two-sided *p* < 0.05 was considered statistically significant.

## 3 Results

### 3.1 Characteristic of participants

As shown in Table 1, Among 164 participates with myocarditis, 51 participants with EF < 55% and 113 participants with EF ≥ 55%. There were no significant differences in age, BMI, or gender among the groups (*P* > 0.05). After adjusting for confounding factors (age, sex, BMI), the EF < 55% group demonstrated a statistically significant decrease in plasma band neutrophil percentage and a significant elevation in HCY concentration (15.48 ± 1.90 μmol/L) compared to the EF ≥ 55% group (*P* < 0.05). However, no statistically significant differences were found in other plasma parameters, including WBC, CRP, HGB, PLT, CK, CKMB, TNI, NT-proBNP, IL-6, folate, VB12, and the segmented neutrophil percentage (*P* > 0.05).

**Table 1:** Demographic characteristics and plasma parameters of pediatric myocarditis patients, grouped by EF < 55% and EF ≥ 55%.

Variables	EF < 55% (*n* = 51)	EF ≥ 55% (*n* = 113)	*P* value
Age (year)	12.33 ± 0.54	12.16 ± 0.34	0.778
BMI (kg/m^2^)	22.73 ± 1.00	21.67 ± 0.51	0.348
Gender			0.905
Male	32 (62.7)	72 (63.7)	
Female	19 (37.3)	41 (36.3)	
Plasma parameters			
IL-6 (pg/mL)	2.72 ± 0.37	2.51 ± 0.26	0.925
HCY (μmol/L)	15.48 ± 1.90	11.45 ± 9.03	0.025
WBC (10^9^/L)	7.01 ± 0.25	6.89 ± 0.21	0.910
Band Neutrophil (%)	51.12 ± 1.97	54.46 ± 1.10	0.048
Segmented Neutrophil (%)	36.96 ± 1.58	35.97 ± 1.03	0.296
CRP (mg/L)	2.50 ± 0.60	2.49 ± 0.57	0.699
HGB (g/L)	143.20 ± 2.13	139.93 ± 1.43	0.170
PLT (10^9^/L)	302.86 ± 8.04	289.80 ± 6.48	0.268
CK (U/L)	162.47 ± 26.37	127.91 ± 10.29	0.122
CKMB (ng/mL)	2.46 ± 0.19	2.49 ± 0.14	0.998
TNI (pg/mL)	32.56 ± 22.38	27.52 ± 15.72	0.903
NT-proBNP (pg/mL)	52.95 ± 8.01	64.84 ± 7.57	0.345
Folate (ng/mL)	7.66 ± 0.62	7.44 ± 0.32	0.458
VB12 (pg/mL)	393.00 ± 23.82	405.00 ± 18.70	0.823

Data was presented as mean ± standard error or *n* (%). *T*-test was used to compare differences in age and BMI; *χ*^2^ test was used to compare differences in gender between groups. General linear model was used to compare differences in plasma parameters, with adjusting for age, gender and BMI. *P* < 0.05 was considered statistically significant. BMI, body mass index; CRP, C-reactive protein; HGB, hemoglobin; PLT, platelet count; IL-6, Interleukin-6; CK, creatine kinase; CK-MB, creatine kinase-MB form; TNI, troponin I; NT-proBNP, N-terminal pro-brain natriuretic peptide; HCY, homocysteine; VB12, vitamin B12.

### 3.2 Association between plasma parameters and the risk of EF<55%

The relationship between plasma parameters and the risk of EF < 55% is shown in Table 2. High HCY concentration demonstrated a statistically positive relationship with the risk of occurrence of EF < 55% [OR = 1.033 (95%CI,1.002–1.064); *P* = 0.034]. The relationship remained statistically significant after adjusting for confounding factors [OR = 1.038 (95%CI,1.003, 1.074); *P* = 0.033]. However, we did not found association between other plasma parameters and the occurrence of EF < 55% (*P* > 0.05).

**Table 2:** Logistic regression analysis of plasma parameters associated with the risk of reduced ejection fraction (EF < 55%) in children with myocarditis.

Parameters	Model 1		Model 2	
	OR 95%CI	*P*	OR 95%CI	*P*
IL-6 (pg/mL)	1.027 (0.914, 1.155)	0.653	1.006 (0.887, 1.141)	0.927
HCY (μmol/L)	1.033 (1.002, 1.064)	0.034	1.038 (1.003, 1.074)	0.033
WBC (10^9^/L)	1.028 (0.880, 1.202)	0.723	1.009 (0.858, 1.188)	0.909
Band Neutrophil (%)	0.979 (0.953, 1.005)	0.117	0.970 (0.941, 1.001)	0.057
Segmented Neutrophil (%)	1.008 (0.978, 1.039)	0.595	1.019 (0.983, 1.057)	0.294
CRP (mg/L)	1.000 (0.943, 1.062)	0.992	0.987 (0.924, 1.054)	0.700
HGB (g/L)	1.014 (0.992, 1.037)	0.204	1.021 (0.992, 1.050)	0.166
PLT (10^9^/L)	1.003 (0.998, 1.008)	0.241	1.003 (0.998, 1.009)	0.263
CK (U/L)	1.002 (0.999, 1.004)	0.158	1.002 (0.999, 1.004)	0.129
CKMB (ng/mL)	0.987 (0.784, 1.244)	0.912	1.000 (0.786, 1.271)	0.998
TNI (pg/mL)	1.000 (0.998, 1.002)	0.855	1.000 (0.998, 1.002)	0.901
NT-proBNP (pg/mL)	0.998 (0.993, 1.003)	0.345	0.997 (0.992, 1.003)	0.351
Folate (ng/mL)	1.016 (0.931, 1.109)	0.720	1.039 (0.942, 1.146)	0.447
VB12 (pg/mL)	1.000 (0.998, 1.001)	0.707	1.000 (0.998, 1.002)	0.820

Model 1 was an unadjusted model.

Model 2 was adjusted for age, gender and BMI.

**Figure 2:**
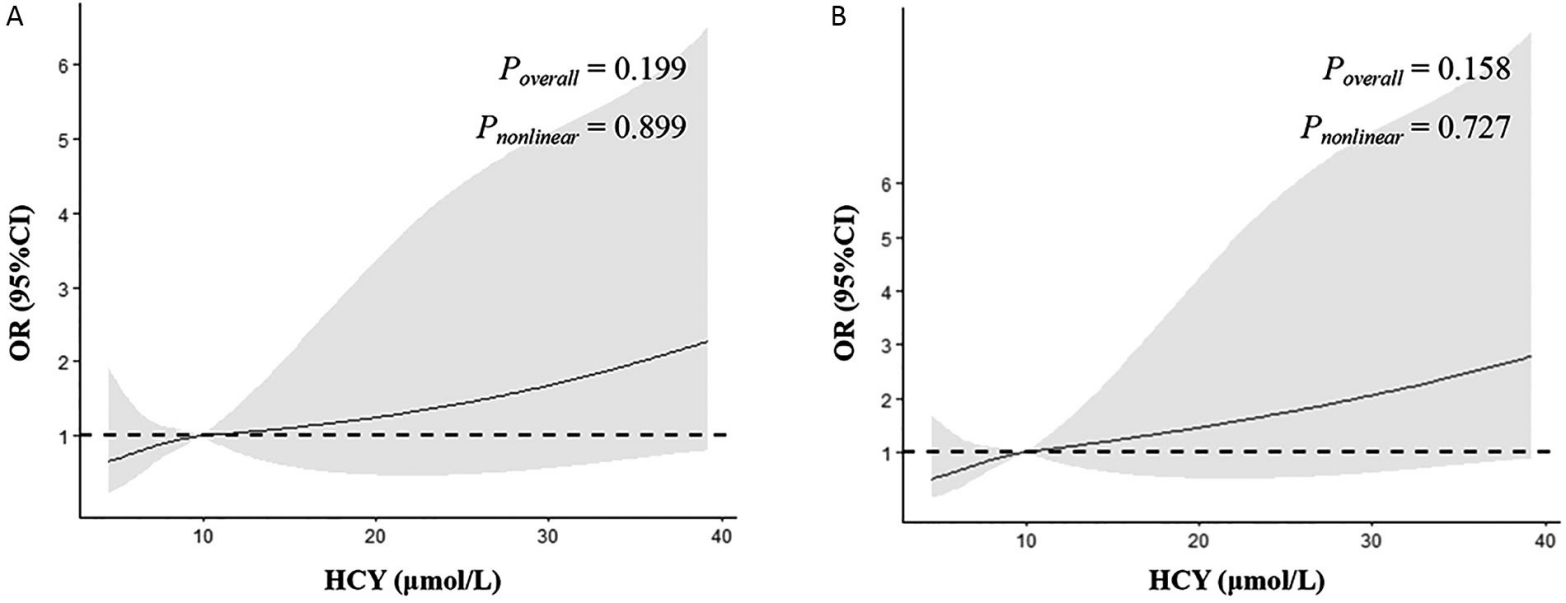
Restricted cubic spline (RCS) analysis illustrating the association between plasma homocysteine (HCY) levels and the risk of reduced ejection fraction (EF < 55%) in children with myocarditis. Odds ratios (ORs) with 95% confidence intervals (CIs) are shown across the range of HCY concentrations. **(A)** Unadjusted model. **(B)** Model adjusted for age, gender, and BMI. Although an increasing trend in risk is observed with higher HCY levels, the association did not reach statistical significance (*P* > 0.05).

We further conducted the RCS plot to investigate the relationship between plasma HCY concentration and the risk of EF < 55%. The RCS results showed that the risk of EF < 55% in children with myocarditis increased with the increase of plasma HCY (Figure 2). However, it was not statistically significant (*P* > 0.05). Suggesting that although a positive trend was observed, the current data do not provide sufficient evidence to confirm a nonlinear or threshold effect of HCY on EF reduction.

### 3.3 Subgroup analysis according to age, gender and BMI

**Figure 3:**
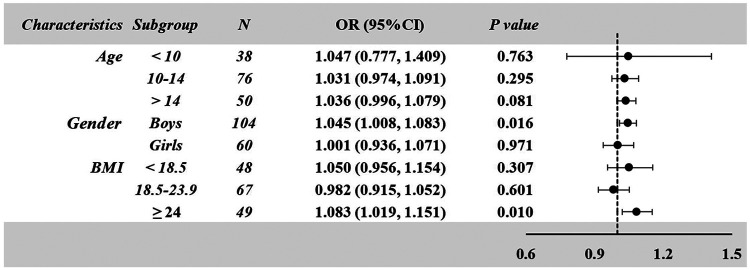
Subgroup analysis of the association between plasma homocysteine (HCY) levels and the risk of reduced ejection fraction (EF < 55%) in pediatric myocarditis patients. Odds ratios (ORs) and 95% confidence intervals (CIs) were estimated using logistic regression models stratified by age, gender, and BMI. A stronger association was observed in male patients and in those with BMI ≥ 24 kg/m^2^.

A subgroup analysis stratified by age, gender, and BMI was conducted, with detailed outcomes presented in Figure 3. It revealed that Higher HCY levels emerged as a significant independent risk factor for reduced EF (EF < 55%), with particularly pronounced associations observed in specific demographic subgroups. This association demonstrated greater significance in boys [OR = 1.045 (95%CI, 1.008–1.083); *P* = 0.016] and individuals with BMI ≥ 24 kg/m^2^ [OR = 1.083 (95%CI, 1.019–1.151); *P* = 0.010].

## 4 Discussion

Myocarditis affects individuals across all age groups, Compared to adults, the incidence of myocarditis in children exhibits a bimodal distribution, infants exhibit a higher incidence, followed by a secondary increase in young adults (26). Two-thirds have a history of preceding viral infections, and over 50% present with fever (10). Viral infection is the primary etiology, with pathogenesis involving direct damage from viral replication in host cells and inflammatory responses mediated by both viral activity and immune reactions (15). The pathophysiology of myocarditis can be divided into three stages: acute viral-induced injury; host innate and adaptive immune responses; and recovery or progression to scarring and dilated cardiomyopathy (15). Compared to adults, the pathophysiological course of myocarditis in children is more rapid, with fulminant myocarditis occurring more frequently than in adults (10). This may stem from the active immune systems of adolescents (27). Therefore, early recognition of myocarditis in pediatric patients is crucial for prognosis.

In this study, we investigated the association between elevated HCY levels and reduced EF in pediatric patients with myocarditis. we observed that elevated HCY levels were a risk factor for patients with EF <55%. This result remained statistically significant after correcting for confounders such as gender, age, and BMI. In previous studies, multiple investigations have extensively demonstrated a negative correlation between HCY and EF, and this association exhibits remarkable consistency across different clinical populations (20, 22–24, 28). This is consistent with the conclusions of the aforementioned studies. This finding demonstrates that elevated HCY levels in pediatric patients with myocarditis are associated with more severe cardiac function impairment. As this is a cross-sectional study, even if we can only establish an association between reduced EF and elevated HCY levels, it still holds significant clinical importance. In the 2024 ACC Expert Consensus Decision Pathway on Strategies and Criteria for the Diagnosis and Management of Myocarditis, reduced EF is recognized as a definitive poor prognostic factor (17). Given the correlation between HCY and reduced EF, early HCY assessment in blood biochemistry can serve as a prognostic reference during the initial treatment phase of myocarditis. This holds significant importance for determining pediatric treatment plans and subsequent follow-up care. Secondly, due to the uncertainty of causality, we cannot definitively determine whether elevated HCY levels during myocarditis result from the inflammatory response, thereby causing reduced EF, or whether individuals with high HCY levels inherently experience poorer outcomes when developing myocarditis. These questions require further clarification through cohort studies.

HCY is an independent risk factor for cardiovascular diseases (4). As a cross-sectional study, although this paper cannot establish a causal relationship between elevated HCY levels and reduced EF in children with myocarditis, we review the potential mechanisms underlying this association based on previous research. HCY has been demonstrated to possess cardiotoxicity, leading to myocardial remodeling and dysfunction (29). The underlying mechanisms involve multiple levels: at the genetic level, HCY decreases the S-adenosylmethionine (SAM)/S-adenosylhomocysteine (SAH) ratio and induces DNA and histone hypomethylation, which alters gene expression and promotes cardiomyocyte hypertrophy (30). At the organelle level, HCY generates reactive oxygen species (ROS), triggers oxidative stress, and impairs mitochondrial protein content and function in cardiomyocytes. HCY also induces endoplasmic reticulum (ER) stress, which leads to Ca^2+^ accumulation, reduced ATP production, decreased membrane potential, and activation of mitosis and apoptosis (30). At the cellular level, HCY induces cardiomyocyte hypertrophy by up-regulating copper-transporting ATPase 1 (ATP7a) protein expression, decreasing copper content, and decreasing cytochrome c oxidase (COX) activity. Interactions between soluble epoxide hydrolase (sEH) and typical transient receptor potential 3 (TRPC3) channels, as well as the calreticulin-nuclear factor of activated T-cells (NFAT) pathway, may also contribute to HCY-mediated myocardial alterations (31–33). Additionally, elevated HCY levels may alter cell communication by regulating the expression of matrix metalloproteinases and connexins in myocardial tissue (34). In this study, patients with EF <55% exhibited higher HCY levels. These mechanisms provide a potential theoretical basis for the association between elevated HCY levels and more severe myocardial injury.

In our study, The RCS results showed an increased risk of EF < 55% in children with myocarditis with increasing plasma HCY. However, this relationship was not statistically significant (*P* > 0.05) due to the small sample size, but this trend still provides some indication of the relationship between HCY and EF. Several factors may explain this finding. First, the overall sample size, particularly the limited number of patients with significantly elevated HCY levels, constrained the statistical power of the RCS analysis. Second, HCY levels in this dataset primarily fell within the upper range of normal values (12.70 ± 0.84 μmol/L), exhibiting a relatively narrow distribution, which may have weakened the observable dose-response effect. Third, reduced EF in acute myocarditis is influenced by multiple interacting factors, including inflammatory activity, myocardial edema, viral infection. The presence of residual confounding factors may also obscure subtle nonlinear relationships. Therefore, the RCS results should be interpreted as hypothesis-generating findings, suggesting a potential risk pattern, requiring further validation through larger prospective studies.

Notably, in the subgroup analysis, the association between high HCY levels and EF < 55% risk showed greater significance in boys OR = 1.045 [95%CI, 1.008–1.083]; *P* = 0.016 and individuals with BMI ≥ 24 OR = 1.083 [95%CI, 1.019–1.151]; *P* = 0.010. In pediatric populations, HCY exhibits a weak correlation with overweight status (35). Previous studies have found elevated levels of total cholesterol and low-density lipoprotein in children with homocystineia. Homocysteinemia is significantly associated with LDL levels (36). The association between elevated HCY levels and overweight appears to be mediated through adipose tissue dysfunction, with the mechanism involving the inhibition of lipolysis through the activation of adenosine monophosphate-sensitive protein kinase (37). MTHFR gene polymorphisms are closely associated with HCY concentrations in obesity (38). However, evidence supporting this mechanistic link in pediatric populations remains insufficient, with only limited epidemiological data showing a conclusive association between hyperhomocysteinemia and excessive obesity in pediatric and adolescent populations (35). In previous studies, boys demonstrated higher HCY levels compared to girls (39), which may be attributed to sex-related differences in amino acid metabolism (40). HCY remethylation rates were found to be higher in females than in males, and this metabolic difference is consistent with sex differences in physiological requirements and utilization patterns of certain amino acids (41). Therefore, close monitoring of plasma HCY levels in overweight male children with myocarditis is essential to mitigate adverse outcomes.

This study has several limitations that should be acknowledged. First, it is a single-center study, which may limit the generalizability of the findings. Second, due to the cross-sectional design, causal inferences between HCY levels and cardiac function cannot be established. Third, the relatively small sample size may reduce the statistical power to detect certain associations. Finally, the absence of follow-up or longitudinal data precludes assessment of dynamic changes in HCY and cardiac function over time. Despite these limitations, our study provides novel insights into the association between HCY and left ventricular EF in pediatric patients with myocarditis. By identifying significant HCY–EF correlations and evaluating potential modifiers such as sex, age, and BMI, this work lays a foundation for future longitudinal studies.

## 5 Conclusion

The aim of this study was to investigate the relationship between HCY and EF in children with myocarditis. The results showed that elevated HCY levels were a risk factor for patients with EF < 55%. In addition, the association between high HCY levels and EF < 55% was more pronounced in two subgroups of patients with BMI ≥ 24 and in boys. HCY has great potential as a biomarker of mycarditis prognosis. Future studies should further investigate whether HCY lowering therapies improve cardiac function and establish a causal relationship between HCY and decreased EF in pediatric patients with myocarditis.

## Data Availability

The raw data supporting the conclusions of this article will be made available by the authors, without undue reservation.

## References

[1] Jakubowski H. Homocysteine modification in protein structure/function and human disease. Physiol Rev. (2019) 99(1):555–604. 10.1152/physrev.00003.2018 30427275

[2] Hermann A Sitdikova G. Homocysteine: biochemistry, molecular biology and role in disease. Biomolecules. (2021) 11(5):737. 10.3390/biom11050737 34063494 PMC8156138

[3] Zaric BL Obradovic M Bajic V Haidara MA Jovanovic M Isenovic ER. Homocysteine and hyperhomocysteinaemia. Curr Med Chem. (2019) 26(16):2948–61. 10.2174/0929867325666180313105949 29532755

[4] Ganguly P Alam SF. Role of homocysteine in the development of cardiovascular disease. Nutr J. (2015) 14:6. 10.1186/1475-2891-14-6 25577237 PMC4326479

[5] Jakubowski H Witucki L. Homocysteine metabolites, endothelial dysfunction, and cardiovascular disease. Int J Mol Sci. (2025) 26(2):746. 10.3390/ijms26020746 39859460 PMC11765536

[6] Lu J Chen K Chen W Liu C Jiang X Ma Z Association of serum homocysteine with cardiovascular and all-cause mortality in adults with diabetes: a prospective cohort study. Oxid Med Cell Longev. (2022) 2022:2156483. 10.1155/2022/2156483 36267812 PMC9578792

[7] Liang Z Li K Chen H Jia J Li J Huo Y The association of plasma homocysteine concentrations with a 10-year risk of all-cause and cardiovascular mortality in a community-based Chinese population. Nutrients. (2024) 16(12):1945. 10.3390/nu16121945 38931298 PMC11206274

[8] Wehner GJ Jing L Haggerty CM Suever JD Leader JB Hartzel DN Routinely reported ejection fraction and mortality in clinical practice: where does the nadir of risk lie? Eur Heart J. (2020) 41(12):1249–57. 10.1093/eurheartj/ehz550 31386109 PMC8204658

[9] Ipek R Holland J Cramer M Rider O. CMR To characterize myocardial structure and function in heart failure with preserved left ventricular ejection fraction. Eur Heart J Cardiovasc Imaging. (2024) 25(11):1491–504. 10.1093/ehjci/jeae224 39205602 PMC11522877

[10] Law YM Lal AK Chen S Cihakova D Cooper LT Jr. Deshpande S Diagnosis and management of myocarditis in children: a scientific statement from the American Heart Association. Circulation. (2021) 144(6):e123–e35. 10.1161/CIR.0000000000001001 34229446

[11] Liu Y Song J Wang W Zhang K Qi Y Yang J Association of ejection fraction with mortality and cardiovascular events in patients with coronary artery disease. ESC Heart Fail. (2022) 9(5):3461–8. 10.1002/ehf2.14063 35866195 PMC9715855

[12] Lam CSP Solomon SD. Classification of heart failure according to ejection fraction: JACC review topic of the week. J Am Coll Cardiol. (2021) 77(25):3217–25. 10.1016/j.jacc.2021.04.070 34167646

[13] Tschope C Ammirati E Bozkurt B Caforio ALP Cooper LT Felix SB Myocarditis and inflammatory cardiomyopathy: current evidence and future directions. Nat Rev Cardiol. (2021) 18(3):169–93. 10.1038/s41569-020-00435-x 33046850 PMC7548534

[14] The Subspecialty Group of Cardiology tSoP, Chinese Medical Association, Collaborating Group of Myocarditis tSGoC, the Society of Pediatrics, Chinese Medical Association, the Editorial Board CJoP, Pediatric Cardiology Committee CCoCP. Chinese Medical doctor association. Diagnostic recommendation for myocarditis in children (version 2018). Chinese Journal of Pediatrics. (2019) 57(2):87–9. 10.3760/cma.j.issn.0578-1310.2019.02.004 30695879

[15] Sagar S Liu PP Cooper LT Jr. Myocarditis. Lancet. (2012) 379(9817):738–47. 10.1016/S0140-6736(11)60648-X 22185868 PMC5814111

[16] Luci H Wei S Lingyun G Yiliang F Fei L Hui X Analysis of the epidemic characteristics and disease burden of hospitalized children with viral myocarditis in China from 2016 to 2021. Chin J Exp Clin Virol. (2024) 2024(04):432–8. 10.3760/cma.j.cn112866-20240219-00020

[17] Writing C Drazner MH Bozkurt B Cooper LT Aggarwal NR Basso C 2024 ACC expert consensus decision pathway on strategies and criteria for the diagnosis and management of myocarditis: a report of the American College of Cardiology solution set oversight committee. J Am Coll Cardiol. (2025) 85(4):391–431. 10.1016/j.jacc.2024.10.080 39665703

[18] Sanguineti F Garot P Mana M O'H-Ici D Hovasse T Unterseeh T Cardiovascular magnetic resonance predictors of clinical outcome in patients with suspected acute myocarditis. J Cardiovasc Magn Reson. (2015) 17(1):78. 10.1186/s12968-015-0185-2 26318624 PMC4553007

[19] Rossi GP Seccia TM Pessina AC. Homocysteine, left ventricular dysfunction and coronary artery disease: is there a link? Clin Chem Lab Med. (2007) 45(12):1645–51. 10.1515/CCLM.2007.353 17990950

[20] Badiou S Dupuy AM Jaussent I Sultan A Mariano-Goulart D Cristol JP Homocysteine as a determinant of left ventricular ejection fraction in patients with diabetes. Clin Chem Lab Med. (2012) 50(6):1099–106. 10.1515/cclm-2011-0851 22706253

[21] Yao Z Li G Li G. Correlation between serum urea nitrogen, cystatin C, homocysteine, and chronic heart failure. Am J Transl Res. (2021) 13(4):3254–61. 34017496 PMC8129353

[22] Nygard O Nordrehaug JE Refsum H Ueland PM Farstad M Vollset SE. Plasma homocysteine levels and mortality in patients with coronary artery disease. N Engl J Med. (1997) 337(4):230–6. 10.1056/NEJM199707243370403 9227928

[23] Cziraki A Nemeth Z Szabados S Nagy T Szanto M Nyakas C Morphological and functional remodeling of the ischemic heart correlates with homocysteine levels. J Cardiovasc Dev Dis. (2023) 10(3):122. 10.3390/jcdd10030122 36975886 PMC10056082

[24] El-Amrousy D Hassan S Hodeib H. Prognostic value of homocysteine and highly sensitive cardiac troponin T in children with acute heart failure. J Saudi Heart Assoc. (2018) 30(3):198–204. 10.1016/j.jsha.2017.11.007 29983495 PMC6026391

[25] Yingjie Z Yarui L. Significance of homocysteine in the occurrence and development of pediatric diseases. Chin J Appl Clin Pediatr. (2019) 34(03):234–7.

[26] Disease GBD Injury I Prevalence C. Global, regional, and national incidence, prevalence, and years lived with disability for 354 diseases and injuries for 195 countries and territories, 1990–2017: a systematic analysis for the global burden of disease study 2017. Lancet. (2018) 392(10159):1789–858. 10.1016/S0140-6736(18)32279-7 30496104 PMC6227754

[27] Ricci JC Farahani NA Davis CJ Ritter KG Parrow LM Tomerlin PI A comparative review of myocarditis in pediatrics versus adults: pathogenesis, diagnosis, and management. Front Immunol. (2025) 16:1601307. 10.3389/fimmu.2025.1601307 41080571 PMC12511060

[28] Gueant-Rodriguez RM Juilliere Y Nippert M Abdelmouttaleb I Herbeth B Aliot E Left ventricular systolic dysfunction is an independent predictor of homocysteine in angiographically documented patients with or without coronary artery lesions. J Thromb Haemost. (2007) 5(6):1209–16. 10.1111/j.1538-7836.2007.02535.x 17403112

[29] Nasir K Tsai M Rosen BD Fernandes V Bluemke DA Folsom AR Elevated homocysteine is associated with reduced regional left ventricular function: the multi-ethnic study of atherosclerosis. Circulation. (2007) 115(2):180–7. 10.1161/CIRCULATIONAHA.106.633750 17200444

[30] Kaplan P Tatarkova Z Sivonova MK Racay P Lehotsky J. Homocysteine and mitochondria in cardiovascular and cerebrovascular systems. Int J Mol Sci. (2020) 21(20):7698. 10.3390/ijms21207698 33080955 PMC7589705

[31] Zhou Y Wang XC Wei JH Xue HM Sun WT He GW Soluble epoxide hydrolase and TRPC3 channels jointly contribute to homocysteine-induced cardiac hypertrophy: interrelation and regulation by C/EBPbeta. Biochim Biophys Acta Mol Basis Dis. (2023) 1869(4):166643. 10.1016/j.bbadis.2023.166643 36669577

[32] Cao Z Zhang Y Sun T Zhang S Yu W Zhu J. Homocysteine induces cardiac hypertrophy by up-regulating ATP7a expression. Int J Clin Exp Pathol. (2015) 8(10):12829–36. 26722473 PMC4680418

[33] Deng Y Li Z An X Fan R Wang Y Li J Hyperhomocysteinemia promotes cardiac hypertrophy in hypertension. Oxid Med Cell Longev. (2022) 2022:1486157. 10.1155/2022/1486157 36046692 PMC9423973

[34] Rosenberger D Gargoum R Tyagi N Metreveli N Sen U Maldonado C Homocysteine enriched diet leads to prolonged QT interval and reduced left ventricular performance in telemetric monitored mice. Nutr Metab Cardiovasc Dis. (2011) 21(7):492–8. 10.1016/j.numecd.2009.11.014 20227264 PMC2889131

[35] de Oliveira Leite L Costa Dias Pitangueira J Ferreira Damascena N Ribas de Farias Costa P. Homocysteine levels and cardiovascular risk factors in children and adolescents: systematic review and meta-analysis. Nutr Rev. (2021) 79(9):1067–78. 10.1093/nutrit/nuaa116 33351941

[36] Youness ER El-Daly SM Abdallah HR El-Bassyouni HT Megahed H Khedr AA Serum homocysteine, lipid profile and BMI as atherosclerotic risk factors in children with numerical chromosomal aberrations. World J Pediatr. (2022) 18(6):443–8. 10.1007/s12519-022-00534-4 35430675

[37] Wang Z Pini M Yao T Zhou Z Sun C Fantuzzi G Homocysteine suppresses lipolysis in adipocytes by activating the AMPK pathway. Am J Physiol Endocrinol Metab. (2011) 301(4):E703–12. 10.1152/ajpendo.00050.2011 21750268 PMC3191546

[38] Fu L Li YN Luo D Deng S Hu YQ. Plausible relationship between homocysteine and obesity risk via MTHFR gene: a meta-analysis of 38,317 individuals implementing Mendelian randomization. Diabetes Metab Syndr Obes. (2019) 12:1201–12. 10.2147/DMSO.S205379 31413611 PMC6662519

[39] Osganian SK Stampfer MJ Spiegelman D Rimm E Cutler JA Feldman HA Distribution of and factors associated with serum homocysteine levels in children: child and adolescent trial for cardiovascular health. JAMA. (1999) 281(13):1189–96. 10.1001/jama.281.13.1189 10199428

[40] Fukagawa NK Martin JM Wurthmann A Prue AH Ebenstein D O'Rourke B. Sex-related differences in methionine metabolism and plasma homocysteine concentrations. Am J Clin Nutr. (2000) 72(1):22–9. 10.1093/ajcn/72.1.22 10871556

[41] Leal AA Palmeira AC Castro GM Simoes MO Ramos AT Medeiros CC. Homocysteine: cardiovascular risk factor in children and adolescents? Rev Assoc Med Bras (1992). (2013) 59(6):622–8. 10.1016/j.ramb.2013.05.004 24182942

